# Small Gold Nanorods: Recent Advances in Synthesis, Biological Imaging, and Cancer Therapy

**DOI:** 10.3390/ma10121372

**Published:** 2017-11-30

**Authors:** Lu An, Yuanyuan Wang, Qiwei Tian, Shiping Yang

**Affiliations:** The Key Laboratory of Resource Chemistry of the Ministry of Education, the Shanghai Key Laboratory of Rare Earth Functional Materials, and the Shanghai Municipal Education Committee Key Laboratory of Molecular Imaging Probes and Sensors, Shanghai Normal University, Shanghai 200234, China; anlu1987@shnu.edu.cn (L.A.); 1000398653@smail.shnu.edu.cn (Y.W.)

**Keywords:** small gold nanorods, seedless, biological imaging, cancer therapy

## Abstract

Over the past few decades, the synthetic development of ultra-small nanoparticles has become an important strategy in nano-medicine, where smaller-sized nanoparticles are known to be more easily excreted from the body, greatly reducing the risk caused by introducing nano-theranostic agents. Gold nanorods are one of the most important nano-theranostic agents because of their special optical and electronic properties. However, the large size (diameter > 6 nm) of most obtained gold nanorods limits their clinical application. In recent years, more and more researchers have begun to investigate the synthesis and application of small gold nanorods (diameter < 6 nm), which exhibit similar optical and electronic properties as larger gold nanorods. In this review, we summarize the recent advances of synthesis of the small gold nanorods and their application for near-infrared light-mediated bio-imaging and cancer therapy.

## 1. Introduction

In recent years, near-infrared light-mediated multifunctional platforms based on inorganic nanomaterials for cancer diagnosis and treatment have been explored widely [1,2,3], including carbon [4,5,6], semiconductors [7,8,9], and noble metals [10,11,12,13]. Gold nanomaterials [10,11,14], especially gold nanorods [15,16,17], attract more attention. Several intrinsic physicochemical properties make gold nanorods a promising multifunctional platform for cancer theranostics. First, the strong surface plasma resonance (SPR) absorption of the gold nanorods enables them to absorb more light even with tiny amounts of gold nanorods [18,19]. Secondly, the SPR absorption of the gold nanorods can be easily tuned to the near-infrared (NIR) window (650–1350 nm) [20,21] where light can penetrate more deeply because of scarce absorption by tissues and blood [22]. Lastly, the gold nanorods can be more easily modified by the thiol compounds via strong Au-S bonds, formed by the thiol group and the surface of gold nanorods [23]. Thus, the design of multifunctional theranostic platforms based on gold nanorods becomes very easy by adding the targeting or imaging agents onto the gold nanorods’ surface [24,25]. These facts—strong absorption, deep penetration, and easy modular functionality—show gold nanorods to be an attractive NIR light-mediated multifunctional platform for cancer theranostics [26,27,28].

Great efforts have been made by the researchers to synthesize the high quality and yield gold nanorods with tunable SPR absorption to investigate the great potential for cancer theranostics [15,29]. However, the commonly synthesized gold nanorods have a scattering cross-section, which is comparable to their absorption cross-section, and which will reduce the photothermal conversion efficiency [30]. On the other hand, these gold nanorods will accumulate in the reticuloendothelial system (RES) organs and tissues rapidly after intravenous injection due to their relatively large size (a width greater than 8 nm and a length of about 40 nm) [31]. Thus, the large-sized gold nanorods cannot be cleared out from the body in a timely manner, causing potential long-term toxicity [32]. Additionally, it is difficult to meet the requirements of the U.S. Food and Drug Administration (FDA), limiting their clinical translation. All these facts will limit the application of large-sized gold nanorods in near-infrared light-mediated bio-imaging and cancer therapy. Fortunately, it was found that the absorption-to-scattering ratio of gold nanorods increases with a decrease in diameter, and gold nanorods with diameters smaller than 10 nm were dominated by absorption, which could minimize the impact of the scattering cross-section [33]. Therefore, small gold nanorods (diameter < 6 nm) which can be quickly excreted from the body need to be developed urgently [34,35].

In order to obtain the small gold nanorods, several different methods have been developed, mainly including the seed-mediated method and seedless method [36,37]. For example, high quality small gold nanorods have been obtained by Wang’s group using the seed-mediated method by changing the seed-to-Au(III) molar ratio in the growth solution [38], while ultra-small size gold nanorods were synthesized by El-Sayed et al. using the seedless method by adjusting the pH and NaBH_4_ concentrations [39]. Due to the excellent properties of the small gold nanorods, including strong absorption, low toxicity and rapid clearance from body, the small size gold nanorods are recently widely used for bio-applications, including photoacoustic imaging, photothermal therapy, and so on. Thus, it is important to review the development of small gold nanorods for the further extending its application.

In this review, we first summarize the recent progress on the synthesis of the small gold nanorods with three different methods. Then, the SPR absorption properties and the surface modification of small gold nanorods were mainly discussed. Finally, we highlight the recent advances of small gold nanorods for a NIR light-mediated multifunctional theranostic platforms, including bio-imaging and cancer therapy.

## 2. Synthesis of Small Gold Nanorods

The synthesis of monodispersed small gold nanorods has attracted much attention for their optical properties and biomedical applications. Many methods have been developed for the synthesis of monodispersed small gold nanorods with different aspect ratios, including the seed-mediated method, the seedless method, and the high-temperature seedless method.

### 2.1. Seed-Mediated Method

The seed mediated method is the typical and more commonly used method for preparing gold nanorods due to the high quality and yield of nanorods, and their tunable size. Generally, two steps are included for the seed-mediated method: the first step is to prepare a small-sized gold seed; the second step is the growth of gold nanorods, which is initiated by the gold seed in the growth solution. The size, aspect ratios, and yield can be tuned by controlling the size of the seed, seed amount, and reaction parameters in the growth solution, including surfactant amount, gold precursor concentration, pH value, and so on.

The seed-mediated growth method was originated in 2001 by Jana et al. [36]. First, the citrate-capped gold seed and growth solution which contained cetyltrimethyl ammonium bromide (CTAB), acetone, hexane, and water, were prepared separately. Then, the growth of the gold nanorods was started by adding the freshly-prepared ascorbic acid to the mixture of gold seed and growth solution in the presence of AgNO_3_. However, the yield of gold nanorods for Jana’s method is very low, and more spherical particles are produced. In 2003, El-Sayed et al. [39] improved Jana’s method by two modifications. One uses the stronger CTAB stabilizer to cap the gold seed; the other introduced silver nitrate to the gold solution before seed addition to facilitate the rod formation and also tune the aspect ratio. For the developed method, the yield of the gold nanorods is about 99% and the aspect ratios can be tuned from 1.5 to 4.5.

**Figure 1:**
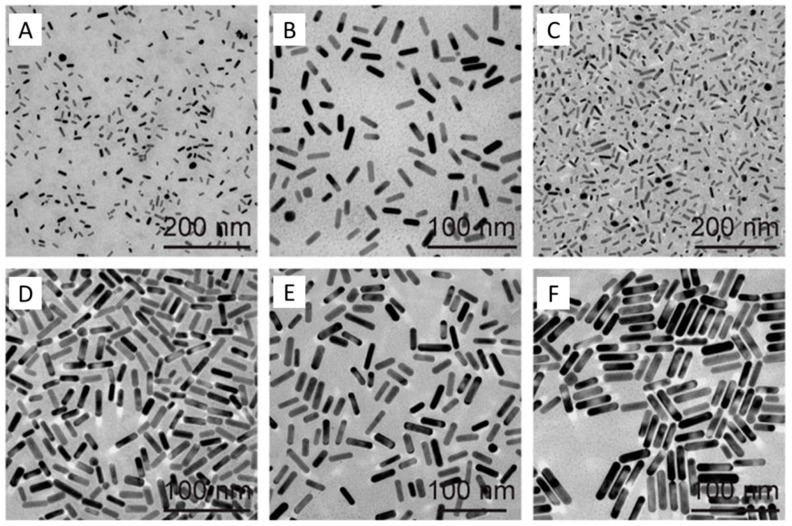
Transmission electron microscopy (TEM) images of the small Au nanorods samples (GmSn) obtained with different molar ratio of seed-to-Au(III) in the growth solution. (**A**–**C**) G1S9, G2S8, and G4S6 were grown with cetyltripropylammonium bromide (CTPAB), and (**D**–**F**) G6S4, G8S2, and G9S1 were grown with CTAB [38].

The seed-mediated method is a typical synthesized method for large size gold nanorods, but it is not very good for Au nanorods smaller than 6 nm. Until now, only several reports use the seed-mediated method to synthesize the small gold nanorods. Murphy’s group [40,41] developed a simple millifluidic reactor which can synthesize the small gold nanorods (diameter ca. 6.6 nm) at the gram-scale, based on the seed-mediated method, but the yield of nanorods and size uniformity are difficult to control. Wang’s group [38] obtained the best quality of small gold nanorods using the seed-mediated method by changing the seed-to-Au(III) molar ratio in the growth solution (Figure 1). They are named GmSn, G, and S, referring to the growth solution and the seed solution, respectively; m is the volume of the surfactant solution used in preparing the growth solution, and n is the volume of the seed solution. CTAB or cetyltripropylammonium bromide (CTPAB) was employed as the stabilizing surfactant in the growth solution. They demonstrate that the molar ratio of the seed-to-Au(III) plays an important effect on the size. When the seed concentration increased in a given growth solution, the size of the obtained gold nanorods will decrease. However, it is difficult to obtain diameters less than 6 nm by this method.

### 2.2. Seedless Method

Recently, the two-step seed growth method has been simplified as a seedless growth method, and is used for the preparation of small-sized gold nanorods (diameter < 5 nm), which is difficult to obtained by the seed-mediated method [42]. For the seedless growth method, no seed preparation step is required for growth of the small-sized gold nanorods, due to nucleation and growth occurring in the same solution. The seed for the growth of the gold nanorods is generated by adding NaBH_4_ directly to the growth solution, which is well known as the strong reducing agent that can reduce Au^3+^ to Au^0^. The small gold nanoparticles formed by adding NaBH_4_ can play the role of seeds to prepare the gold nanorods [43,44]. By this seedless method, the ultra-small gold nanorods (diameter < 5 nm) will be easily obtained because the directly-formed seed is small enough in the growth solution [37].

**Figure 2:**
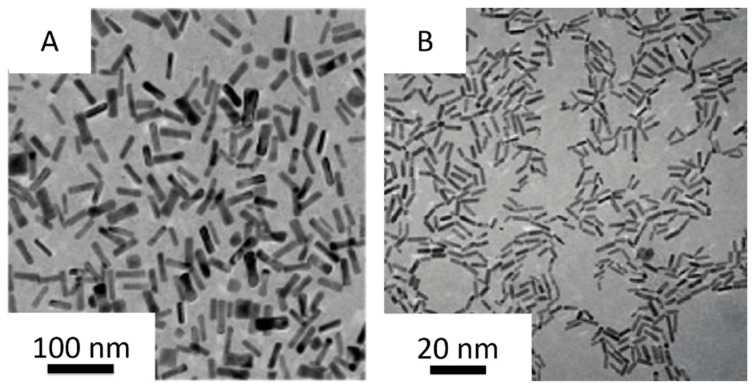
Typical TEM images of the small gold nanorods obtained by Jana et al. [42] ((**A**) 1 mM HAuCl_4_, 0.2 M CTAB, 0.2 mM AgNO_3_, 2 mM ascorbic acid, 0.25 μM BH_4_), and El-Sayed et al. [37] ((**B**) 5.0 mL HAuCl_4_, 5.0 mL CTAB, 270 μL AgNO_3_, 8 μL HCl, 70 μL ascorbic acid, 15 μL NaBH_4_).

The seedless method was discovered by Jana et al. firstly (Figure 2A) [42]. In the CTAB micellar solution of the HAuCl_4_, the strong (NaBH_4_) and weak (ascorbic acid) reducing agents were introduced, in which the CTAB micelle was the template for nanorod growth, strong reducing agent was used to generate the seed in the growth solution directly and the weak reducing agent helped the nanoparticles to grow. They found that if the nucleation kinetics of nanoparticle formation are properly adjusted, the elongated rod-like micelle surface can be a useful template, and the resulting nanoparticles would be highly anisotropic and near-monodisperse. However, the nucleation kinetics of nanoparticle formation is difficult to control and the preparation process is accompanied by a large number of spherical gold nanoparticles, resulting in a low yield of gold nanorods. El-Sayed et al. [37] further developed the seedless method (Figure 2B). They found that the pH plays a crucial role in the monodispersity of the nanorods when the NaBH_4_ concentration of the growth solution was adjusted to control the reduction rate of the gold ions. The reducing power of ascorbic acid and NaBH_4_ decreases with decreasing pH, and the homogeneity of the small gold nanorods increased. At the optimized pH and NaBH_4_ concentrations, smaller gold nanorods were produced by adjusting the CTAB concentration in the growth solution. The higher concentration of CTAB in the growth solution stabilized initial single crystalline nuclei and decreased the growth of rods more than usual, so the gold nanorods were smaller compared to those prepared at a lower CTAB concentration. In addition, the concentration of silver ions in the growth solution was found to be pivotal in controlling the aspect ratio of the nanorods. The aspect ratio decreases as the silver ions concentration decreases. By this new method, it is easier to prepare higher yield, high quality, and ultra-small gold nanorods.

### 2.3. High-Temperature Seedless Method

**Figure 3:**
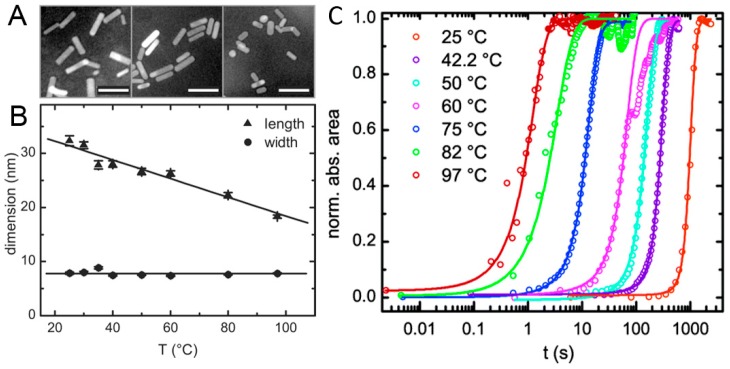
(**A**) TEM images obtained from gold nanorods synthesized at 25 °C, 50 °C, and 97 °C from left to right. The scale bars indicate 50 nm. (**B**) Particle dimension as obtained from TEM analysis. The error bars represent the error in the mean value of the distribution of the respective dimension. (**C**) Evolution of the integrated absorbance vs. time for nanorods synthesized at different temperatures. The solid lines are sigmoidal fits to the experimental data points [44].

Many groups have reported methods for synthesizing gold nanorods at room temperature. Perez-Juste et al. [45] indicated that reaction temperatures close to room temperature are more beneficial for higher nanorod yields. However, Zijlstra et al. [44] synthesized gold nanorods at temperatures varying between 25 and 97 °C, and presented a kinetics study of seedless nanorod synthesis at high temperatures (Figure 3). They found a decrease in rod length when the temperature was gradually raised to 97 °C. It demonstrates that three orders of magnitude increase in the growth rate for Au nanorods synthesized at 97 °C and an average activation energy for growth on all facets to be 90 ± 10 kJ mol^−1^. High-temperature gold nanorod synthesis opens the door to resolving two important issues which have not been addressed in the literature so far. First, ultrafast high-temperature synthesis presents a better system for rapid production of gold nanorods for potential commercial applications. Second, the fact that gold nanorods form at high temperatures suggests that using a thermally-activated reducing agent is possible. Most reports on gold nanorod synthesis utilize NaBH_4_ to initiate the formation of gold nanorods [39]. NaBH_4_ reacts with water and has to be used immediately after preparation, which compromises reproducibility. Using a thermally-activated reducing agent would avoid the use of the unstable NaBH_4_, resulting in stock growth solutions that are stable at room temperature.

## 3. Surface Modification of Small Gold Nanorods

Even though CTAB is an almost necessary surfactant for the synthesis of the gold nanorods, the high cytotoxity of CTAB limited its application in biochemistry and biomedicine [46]. Thus, the CTAB must be removed from the surface of the gold nanorods before it is used for bio-applications. Until now, several strategies have been investigated for solving these problems [18,47,48,49,50,51,52]. Among of them, coating organic or inorganic materials on Au nanorods and replacing the CTAB by thiol-terminated molecules have been proven to be the most effective approaches for improving their biocompatibility.

### 3.1. Surface Coating Method

For the surface coating method, SiO_2_ or polymers (e.g., bovine serum albumin) were normally used to directly coat on the surface of gold nanorods. Due to the effective and high hardness of SiO_2_ coating, the gold nanorods coated with SiO_2_ can not only reduce the toxic effect, but also prevent them from aggregating. In addition, the pores are generated in the SiO_2_ coating, and the SiO_2_-coated gold nanorods can also be used for drug delivery [47]. Bovine serum albumin (BSA) is one kind of low cost biomacromolecules and is widely used for biomedicine. In order to improve the biocompatibility of the gold nanorods, BSA has also been used to coat the surface of the gold nanorods. Due to strong thiol binding sites on the BSA, the gold nanorods are easily been coated by the BSA when they mix together. It was easily demonstrated if the BSA was coated on the gold nanorods by the extinction spectra, the absorption maxima of the gold nanorods showed a distinct redshift after being covered by BSA. It have been demonstrated that BSA-coated small gold nanorods exhibit better biocompatibility [48].

### 3.2. Ligand Exchange Method

**Figure 4:**
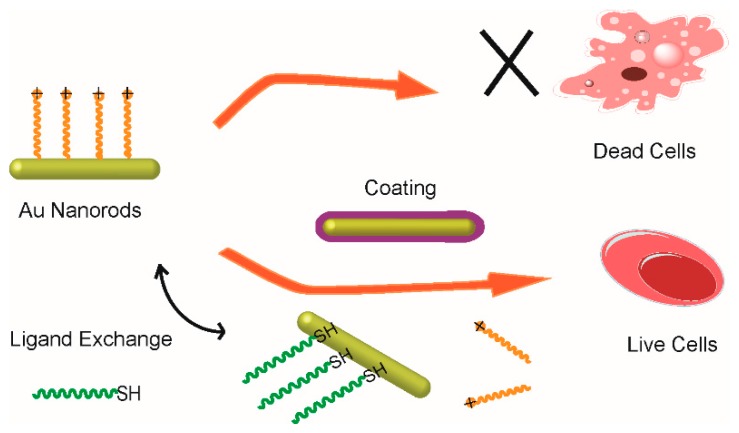
Schematic illustration of the surface modification of small gold nanorods by surface coating and ligand exchange methods.

The ligand exchange method is another commonly used method to remove the CTAB on the surface of the gold nanorods. For this method, thiol-terminated molecules, such as 11-mercaptoundecanoic acid and thiol-terminated polyethylene glycol (SH-PEG) are used to replace the CTAB due to the strong Au-S covalent bond. Several studies [49,50] have demonstrated that 11-mercaptoundecanoic acid can replace the CTAB on the Au nanorods effectively. The thiol of the 11-mercaptoundecanoic acid can bind on the Au nanorods firmly via the Au-S bond, while the carboxyl of the 11-mercaptoundecanoic acid can be used to conjugate with other biomolecules, which is beneficial to the application of gold nanorods in biomedicine fields. However, the low water content of the 11-mercaptoundecanoic acid capped gold nanorods limits its wide usage in bio-applications. Due to the ability of PEG to prevent undesired protein adhesion while at the same time being nontoxic and having good water solubility [51], the thiol-terminated polyethylene glycol (SH-PEG) with functional group (–NH_2_ or –COOH) has been widely used in the surface modification of gold nanorods. As shown in Figure 4, the CTAB can be removed completely once the SH-PEG is added to the CTAB-capped gold nanorods solution via ligand exchange. Several reports have demonstrated that the PEG-capped gold nanorods can improve biocompatibility effectively and they have been used as imaging and photothermal therapy agents [18,52].

## 4. Biological Imaging

In the field of non-invasive diagnostic and therapeutic fields for cancer, real-time imaging of cancer is a goal that people have been pursuing [53,54,55]. Fluorescence imaging as a pure optical imaging technology has been widely used for cancer detection [56,57,58]. Even though the sensitivity of fluorescence imaging is very high, most of the fluorescence sensor is based on ultra-violet-visible (UV-VIS) light [59] and the low penetration depth limits their applications in vivo. Therefore, it is necessary to find a high-contrast and high-resolution non-destructive medical imaging method. 

### 4.1. Photoacoustic Imaging

Photoacoustic tomography (PAT), which is based on the NIR laser, developed quickly recently as a non-destructive medical imaging method [60,61,62], which combines the high contrast characteristics of optical imaging and the high penetration depth characteristics of ultrasound imaging [63,64,65]. Photoacoustic (PA) imaging agents that show strong NIR absorption can effectively improve the contrast and also be investigated widely by the researchers [62]. Among all of the photoacoustic agents including organic dyes [66], semiconductors [67,68], and noble metal materials [69,70], gold nanorods are the most widely used as the NIR absorption can be precisely regulated by adjusting the aspect ratio.

**Figure 5:**
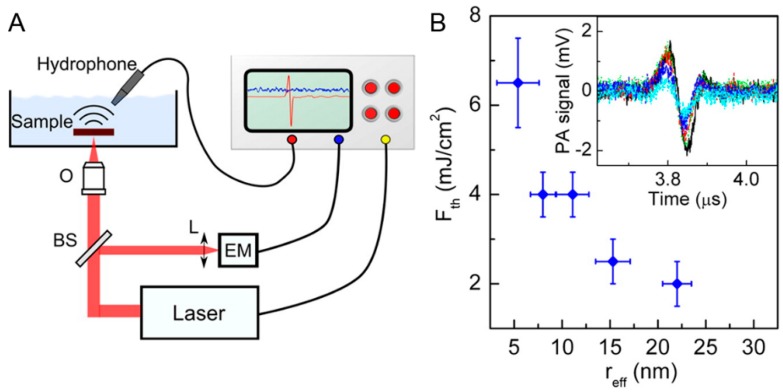
(**A**) Sketch of the setup used for the photoacoustic experiments (O, objective; L, focusing lens; BS, beam splitter; EM, energy meter); (**B**) trend of *F*_th_ as a function of effective nanoparticle radius (r_eff_). In the inset: comparison of PA response with a single pulse excitation at fluence *F* < *F*_th_ for samples containing gold nanorod (GNR)5 (black line), GNR8 (green line), GNR11 (red line), GNR15 (dark blue line), and GNR22 (light blue line). GNR5, GNR8, GNR11, GNR15, and GNR22, with the numbers denoting their average effective radii (radius of a sphere having the same volume as the rod) in nanometers [71].

Pini et al. [71] investigate the influence of size on the photostability and reproducibility of photoacoustic conversion of gold nanorods embedded in biomimetic phantoms. They tested photostability of different sized Au nanorods by acquiring the PA response at the level of single laser shots (Figure 5). PA signals with good signal-to-noise ratios were recorded from all samples at fluences below the maximal permissible exposure limits. Within this test, Au nanorods suffered from partial reshaping and sublimation or fragmentation, which changed their plasmon bands and limited their value as a PA contrast agent. However, there is an interesting phenomenon in that smaller nanoparticles provides better stable signals and have tolerate higher fluencies (Figure 5B). These results provide new inspiration and indications for small Au nanorods for specific PA applications in biomedical imaging. Subsequently, Song et al. [31] developed a small gold nanorods (AuNR) vesicles (≈60 nm in size) coated with polyethylene glycol (PEG) and poly(lactic-co-glycolic acid) (PLGA) as a PA imaging agents. In comparison with the PEGylated AuNR, the mice treated with the same amount of the AuNR@PEG/PLGA vesicles showed a much stronger PA signal in the tumor region at the same time points, suggesting higher uptake of the AuNR@PEG/PLGA vesicles in the tumor region.

### 4.2. Two-Color Photothermal Imaging Microscopy

**Figure 6:**
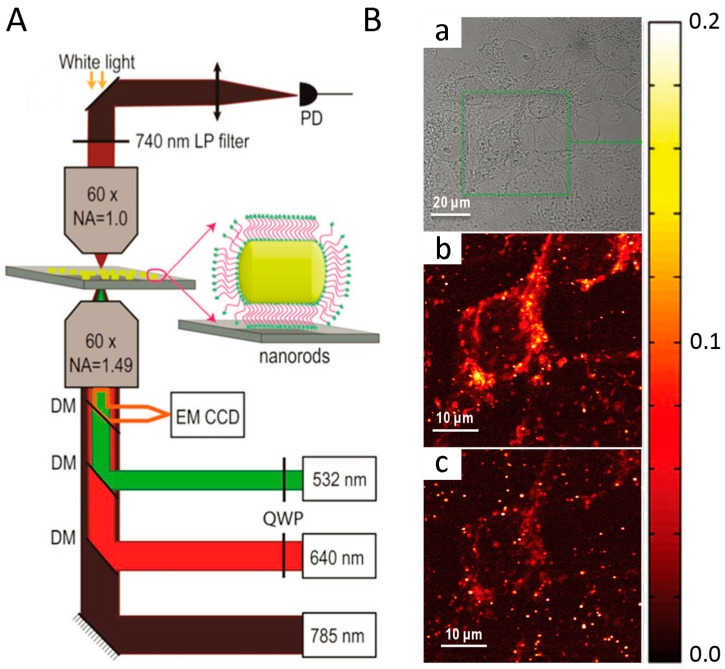
(**A**) Schematics of the two-color photothermal imaging microscopy with a near infrared probe beam at 785 nm and excitations beam at 532 or 640 nm; (**B**) White light (**a**) and PhI images of COS 7 cells incubated with nanorods under (**b**) 532 and (**c**) 640 nm excitation. Photothermal imaging microscopy recorded under red excitation shows very weak mitochondrial background signals compared to those acquired under green excitation [78].

Photothermal imaging (PhI) microscopy technology displays extremely stable signals and has unprecedented sensitivities for detecting tiny absorbers with an absorption cross-section as small as a few 10^−16^ cm^2^ [72,73,74]. Several types of nanoparticles including gold nanoparticles, carbon nanotubes, and quantum dots [75,76] have been studied for ultrasensitive photothermal imaging applications. However, these nanoparticles have to be excited at their plasmon resonance at around 530 nm which is similar with background signal from endogenous cellular components. The use of gold nanorods as small probes absorbing in the near infrared is a promising strategy for single-particle level detection, as they would combine good subcellular accessibility, low contribution from intrinsic cellular signals, and perfect photostability [77]. Concerning this, Lounis et al. [78] developed a new strategy for photothermal imaging based on small gold nanorods. Photothermal imaging microscopy (Figure 6A) is constructed with a two-color excitation beam and a near infrared probe beam that can resonate the nanorods in its transverse or longitudinal plasma resonance. Due to the strong optical absorption tunable from the red to the near infrared, the use of small gold nanorods based on this imaging technology can minimize background signals from the cell organelles. As shown in the Figure 6B, the cellular (mitochondrial) structures are clearly visible under 532 nm excitation (Figure 6B(b)), which complicates the identification of nanorods around the mitochondria at this excitation wavelength. By contrast, background signals originating from mitochondria are notably reduced under 640 nm excitation (Figure 6B). In addition, individual nanorods display notably higher PhI signals under 640 nm excitation compared with 532 nm excitation, facilitating their detection in cellular environments. This small gold nanorod-based photothermal imaging microscopy technology will constitute next generation photothermal probes for studying complex molecular dynamics in biological systems owing to their small size, tunable NIR-absorption, absolute photostability, and chemical suitability for surface functionalization and bioconjugation.

### 4.3. NIR-Absorbing Imaging

**Figure 7:**
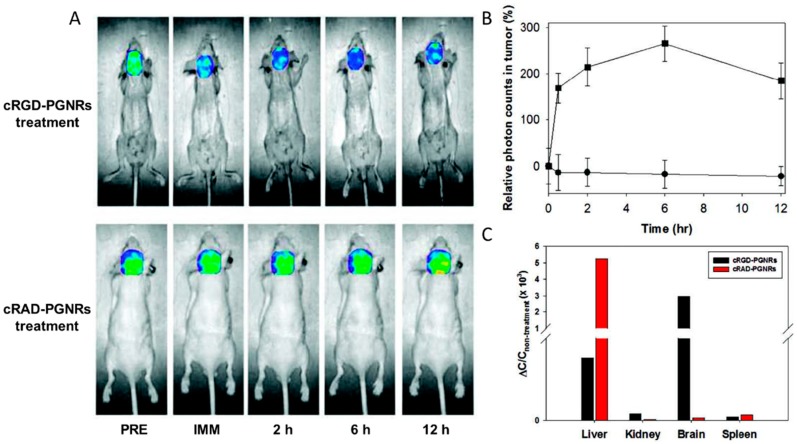
In vivo non-invasive near-infrared (NIR) absorption images of real-time tumor specificity of cRGD-PGNRs. (**A**) In vivo time-dependent brain region biodistribution of cRGD-PGNRs and cRAD-PGNRs as a control; (**B**) relative photon counts of in vivo tumor target specificity of cRGD-PGNRs (square) and cRAD-PGNRs (circle) was recorded; and (**C**) relative quantification of in vivo biodistribution of cRGD-PGNRs and cRAD-PGNRs in different tissues [81].

NIR light (700–1000 nm) for NIR optical imaging [79] can penetrate several centimeters into tissue, because hemoglobin and water, the primary absorbers of visible and infrared light, experience their lowest absorptions in the NIR region. Thus, NIR-absorbing imaging could offer a potentially non-invasive and real-time characterization method for disease using NIR imaging probes [80]. Among the reported NIR imaging probes, including quantum dots, fluorescent dye-doped nanoparticles, etc., gold nanorods are a potential direct NIR absorption imaging probe because the main absorption band is located in the NIR region due to longitudinal surface plasmon. Haam et al. [81] functionalized the Au nanorods with cyclic Arg-Gly-Asp peptides (cRGD). Figure 7 shows the selective NIR-absorbing imaging using cRGD-conjugated PEGylated GNRs (PGNRs). After intravenously injecting with the cRGD-conjugated PGNRs and cRAD-conjugated PGNRs into mice with orthotopic glioma xenografts (*n* = 4), the mice were imaged by NIR absorption imaging for 12 h. Consequently, specific targeting of cRGD-PGNRs to the tumor region was observed via a significant increase in the absorption signal (high absorbance, blue color) that was maintained for 12 h. However, when control cRAD-PGNRs were injected into the tumor-bearing mouse model, the absorption at the tumor site did not change for 12 h. This method is more efficient and simple to determine the localized surface plasmon resonances (LSPR) absorption intensity in molecular imaging. 

## 5. Cancer Therapy

### 5.1. Photothermal Therapy

NIR laser-driven photothermal therapy, which converts NIR laser energy to heat energy, has attracted much interest due to its minimally invasive and potentially effective results compared with the conventional approaches, such as surgery, radiation therapy, chemotherapy, hormone therapy, immunotherapy, etc. [3,82]. In order to promote the photothermal conversion efficiency and particularly improve laser discrimination for targeted cancers, the photothermal agents are generally indispensable [83,84,85]. Among various photothermal therapy agents, the strong absorption properties of the gold nanorods from the visible region to the near-infrared region allows light energy to be efficiently converted to thermal energy under near-infrared laser irradiation, making it possible to perform laser-selective heating at a local range [52,86]. Moreover, the gold nanorods with diameters smaller than 10 nm are dominated by absorption, which could minimize the impact of the scattering cross-section [30,33]. Thus, the small gold nanorod-assisted laser thermal method has great applications in bio-imaging and cancer therapy, which can selectively destroy cancer cells and not damage benign cells [87,88].

**Figure 8:**
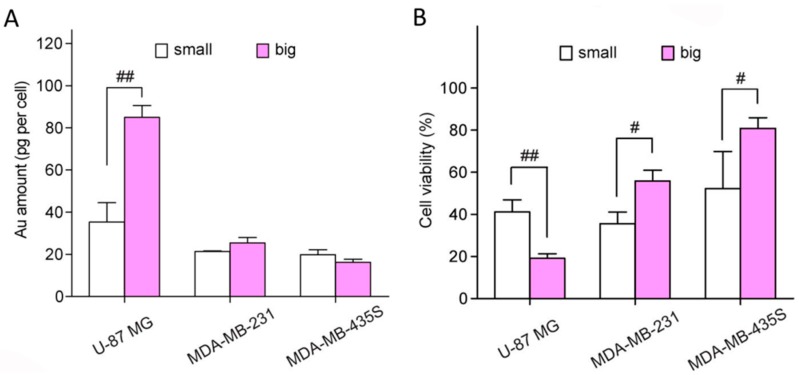
(**A**) Intracellular Au contents of the small (white) and large (pink) silica-coated Au nanorods samples in U-87 MG, MDA-MB-231, and MDA-MB-435S cells; and (**B**) cell viability upon photothermal therapy with small (white) and large (pink) silica-coated Au nanorod samples in U-87 MG, MDA-MB-231, and MDAMB-435S cells [38].

Utilizing the prepared absorption-dominant small gold nanorods, Jia et al. [38] compared their photothermal performance with larger-sized gold nanorods. The cellular uptake efficiencies of the two nanorods samples in three cell lines (U-87 MG, MDA-MB-231, and MDA-MB-435S cells) were evaluated by inductively coupled plasma optical emission spectrometer (ICP-OES) in comparison to addition of the same concentration of Au. They found the internalized number of large Au nanorods was much larger than that of small nanorods in U-87 MG lines. However, both samples showed similar cellular uptake abilities in MDA-MB-231 and MDA-MB-435S cell lines (Figure 8A). These results indicate that both the particle size and cell type influence the cellular uptake of gold nanorods. Subsequently, the photothermal performance was performed on three different cell lines under the irradiation of 809 nm laser with a power density of 12 W·cm^−2^ for 3 min, then evaluated and compared by 3-(4,5-dimethylthiazol-2-yl)-2,5-diphenyltetrazolium bromide (MTT) assay (Figure 8B). The photothermal therapy (PTT) efficiency per unit amount of the internalized Au nanorods was defined as the cell viability reduction divided by the intracellular Au content in each cell line. Compared with the values of 0.95, 1.7, and 1.2% per pg of Au in U-87 MG, MDA-MB-231, and MDA-MB-435S cells of large gold nanorods, the absorption-dominant small gold nanorods exhibit much higher values (1.7%, 3.0%, and 2.4%). These results demonstrate that the small Au nanorods show a higher photothermal therapeutic efficacy on these cancer cells than the large Au nanorods at the same internalized Au amount, and suggest that the absorption-dominant small Au nanorods are promising for plasmonic photothermal conversion-based biomedical applications.

**Figure 9:**
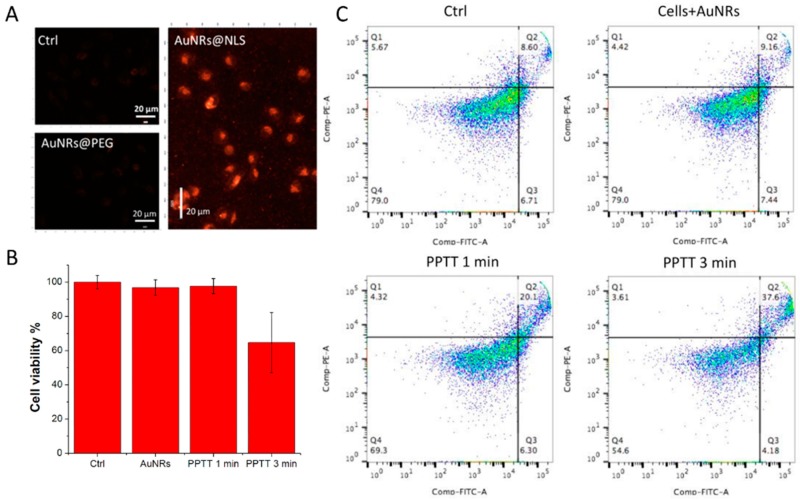
(**A**) Dark-field images of pure HSC-3 cells, cell incubated AuNRs@PEG, and cells incubated in AuNRs@NLS for 24 h. Scale bar = 20 μm; cell viability (**B**) and apoptosis/necrosis assay (**C**) for the HSC-3 cells treated with PPTT at different times; Q1 (necrosis), Q2 (apoptosis), Q3 (early apoptosis) and Q4 (early apoptosis) [86].

El-Sayed et al. [86] synthesized the small gold nanorods (average size: ~25 nm × 6 nm) functionalized with methoxy polyethylene glycol thiol (mPEG-SH), Arg-Gly-Asp (RGD) peptides and nuclear localization signal (NLS) peptides. The uptake of gold nanorods was observed through dark-field (DF) microscopy (Figure 9A). Human oral squamous cell carcinoma (HSC-3) cells were incubated with AuNRs of 2.5 nm for 24 h. Compared with pure cells and cells incubated AuNRs without NLS, clearly internalization was observed by DF microscopy for cells exposed to AuNRs-NLS. The effect of plasmonic photothermal therapy (PPTT) was confirmed by cell viability assays and apoptosis/necrosis assays (Figure 9B,C). A 808 nm NIR laser with power of 5.8 W/cm^2^ was used to irradiate the cells at different times. Compared with cells without laser irradiation, and cells only incubated with AuNRs-NLS, the AuNRs-NLS with laser groups has an obvious effect of PPTT after exposure to the laser for 3 min. The percentage of viability for the HSC cells incubated with AuNRs-NLS after laser irradiation decreased to 60%, and the number of apoptotic cells also increased. These results indicated that the AuNRs-NLS can accurately target the nucleus and enhance plasmonic photothermal therapy.

### 5.2. Image-Guided Photothermal Therapy

**Figure 10:**
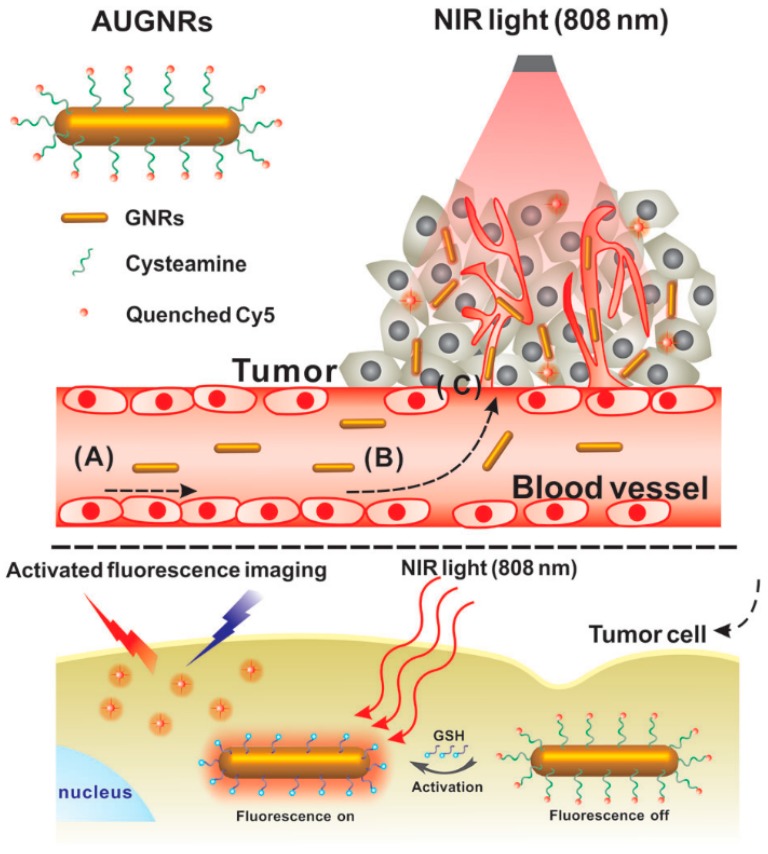
Schematic illustration of the whole procedure for the activatable ultrasmall GNR-based “off–on” fluorescence imaging-guided PTT in tumor cells [28].

Recently, theranostic nanomaterials for real-time diagnosis and cancer PTT has been an attractive method for the treatment of solid tumors as it has the advantages of high efficiency, concurrent accurate diagnosis and efficient in situ therapy of tumors [89]. In this regard, absorption-dominant small-sized gold nanorods (GNR) with diameters smaller than ~6 nm have been investigated for photo-activated cancer therapy. To make the GNR-based PTT visualization, various imaging agents were employed to be integrated with GNRs. However, these imaging-guided therapy patterns still suffer from a low signal to noise ratio [90]. Based on this background, Zhang et al. [28] successfully fabricated an original activatable theranostic agent (AUGNRs) for “off–on” fluorescence imaging guiding PTT (Figure 10). The CTAB-coated ultrasmall GNRs were first placed in cysteamine, and a near-infrared dye (Cy5) conjugated onto the ultrasmall gold nanorods as the fluorescent component. Cy5 was highly quenched by the GNRs in a normal tissue, while being activated in the tumor cells. For the existence of glutathione (GSH), a highly reactive thiol were found in the cytoplasm of tumor cells. GSH can competitively replace the Cy5 and conjugate with the gold nanorods, and the fluorescence of Cy5 can recover rapidly. The study provided a new strategy for clinical tumor theranostics with image-guided photothermal cancer therapy.

**Figure 11:**
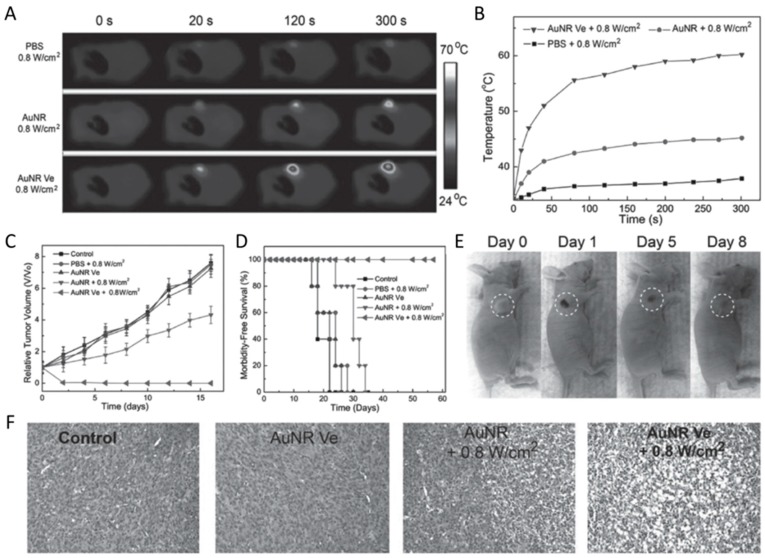
In vivo photothermal ablation of tumor after intravenous injection of Au nanorod vesicles followed by laser irradiation. (**A**) Infrared thermographic maps and (**B**) temperature changes of the tumor region treated with small AuNRs and AuNR Ve and irradiated with a 808 nm laser at different power densities; (**C**) tumor growth curves and (**D**) survival curves of tumor-bearing mice treated with phosphate-buffered saline (PBS), small AuNRs and AuNR Ve and laser irradiation; (**E**) photographs of the tumor-bearing mice at days 0, 1, 5, and 8 d after being treated with the AuNR Ve; and (**F**) hematoxylin and eosin (H&E) staining of the tumor tissue after different treatments [31].

Concerning the balance of higher tumor accumulation efficiency and rapid clearance from the body after therapy [91,92,93], a vesicle assembled by ultra-small gold nanorods was developed by Chen et al. [31] to improve the photothermal therapeutic effect (Figure 11). As shown in Figure 11B, the temperature of the tumor for the mice injected with AuNR@PEG/PLGA vesicles (AuNR Ve) increased up to 20 °C after 5 min of irradiation with an 808 nm laser (0.8 W cm^−2^), which was much higher than the mice treated with AuNR@PEG (AuNR, ≈5 °C temperature increase) and phosphate-buffered saline (PBS) (negligible temperature increase). The higher temperature will induce irreversible tissue damage, which is necessary for the photothermal therapy. The tracked curative effect (Figure 11D) further supports this conclusion, as all the tumors were completely ablated and no reoccurrence was observed when treated with AuNR Ve with a 808 nm laser, compared with the AuNR and laser irradiation group. The tumor sections stained with hematoxylin and eosin for the AuNR Ve plus laser-treated group showed an intensive necrosis area, while highly pleomorphic nuclei and many mitoses, which are the features of the infiltrating tumor cells, was observed for the PBS or laser-only treatment group. Most importantly, most of the vesicles were cleared from the body after ten days post-injection, due to most of the vesicles being disassembled into single polyethylene glycol-modified Au nanorods as triggered by the hydrolysis of PLGA, which is very essential and beneficial for meeting the requirements of the US Food and Drug Administration [94]. These results suggest that the newly-developed ultra-small gold nanorod vesicles provide opportunities for further clinical translation. 

### 5.3. Cell-Mediated Photothermal Therapy

**Figure 12:**
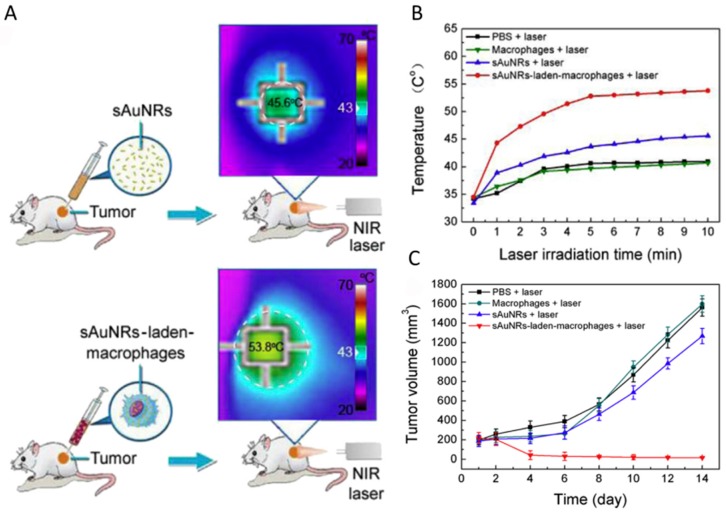
(**A**) Diagram highlighting the difference between the treatment of free small gold nanorods and macrophage-loaded small gold nanorods; (**B**) temperature profile of tumor under 808 nm light irradiation for 10 min; and (**C**) growth of tumors in the different groups of mice after the irradiation treatments [99].

In order to overcome the drawback that the injected nanoparticles cannot penetrate the tumor mass, leading to incomplete ablation and disease recurrence [95], the cell-mediated delivery of nanoparticles, which can cross the nearly-impermeable biological barriers to reach many areas in the body [96,97,98], was developed to improve agent delivery in vivo and enhance photothermal agent efficiency. Based on this, the macrophage delivery system was used by Chu et al. [99] to transport 7 nm diameter Au nanorods for cancer therapy (Figure 12). They first investigated macrophage uptake, which is important for photothermal conversion. Compared with the commonly used 14 nm diameter gold nanorods, the small gold nanorods showed much higher macrophage uptake and negligible cytotoxicity due to their small size. Then, the photothermal therapeutic effect was studied by intratumoral injection of 50 μL of PBS (control), free small gold nanorods (105 μg Au) dispersed in 50 μL of PBS, or small gold nanorod-laden macrophages (105 μg Au in ~1 × 10^6^ RAW264.7 macrophages) dispersed in 50 μL of PBS. The macrophages could deliver small gold nanorods to the entire tumor after intratumoral injection, resulting in photothermal conversion being greatly improved almost everywhere in the tumor, with tumor recurrence rates minimized compared to free BSA-coated small gold nanorods. Their findings not only provided an effective approach to improving photothermal therapy efficiency by delivering the agents to whole tumors, but also expedited the clinical application of nanotechnology for cancer treatment.

### 5.4. Photothermal-Chemo Combination Therapy

**Figure 13:**
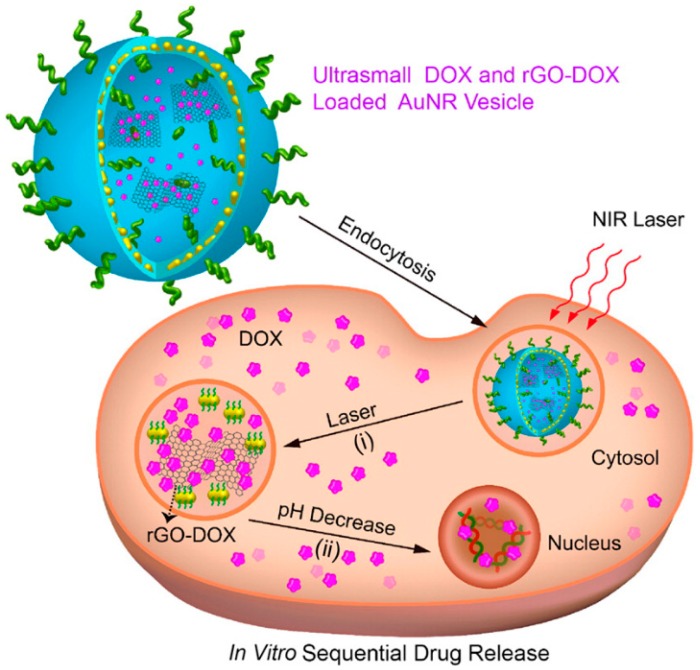
Schematic illustration of sequential DOX release triggered by (**i**) remote NIR laser irradiated photothermal effect and (**ii**) acidic environment of the cancer cell [102].

Reduced graphene oxide (rGO) nanoparticles with a large surface area for drug loading and photothermal effects for photothermal therapy have been widely explored for theranostic applications [100]. Although rGO can absorb light from the UV to NIR and subsequently release it as heat by nonradioactive decay, the broad absorption spectrum and low quantum efficiency of rGO means that it has relatively low photothermal conversion efficiency [101]. rGO-conjugated doxorubicin (DOX) also led to potential toxicity while circulating in a physiological environment. In a recent study, a new kind of carbon-metal hybrid rGO-conjugated DOX (rGO-DOX)-loaded ultrasmall plasmonic gold nanorod vesicle (rGO-AuNRVe-DOX) (Figure 13) for integrated chemo-photothermal therapy was successfully fabricated by Chen et al. [102]. The gold nanorod vesicle was prepared by assembling amphiphilic small gold nanorods (~9 nm × 2 nm) grafted with poly-(ethylene glycol) (PEG) and poly(lactic-co-glycolic acid) (PLGA). The nanorod vesicle can avoid rGO-DOX to interact with normal tissue and also enhance the photothermal effect. Additionally, the inside of a plasmonic metal shell can behave as a cavity where electromagnetic radiation is concentrated, leading to increased light absorption efficiency of the encapsulated rGO [103]. Furthermore, the nanovesicles will break and release the rGO-DOX and DOX from the vesicle, improving cancer therapy efficacy. 

## 6. Cytotoxicity and Metabolizable Ability of Small Gold Nanorods

**Figure 14:**
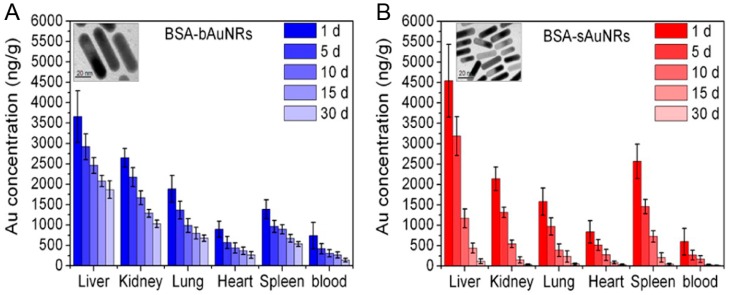
In vivo biodistribution and clearance of (**A**) BSA-bAuNRs and (**B**) BSA-sAuNRs together with Au concentrations at the different time points of 1, 5, 10, 15, and 30 days after intravenous injection (5 mg Au/kg). The inset are typical TEM images of the bAuNRs and sAuNRs, respectively [34].

The safety profile of gold nanorods remains largely undefined. Generally speaking, it is considered biocompatible. Several studies [104,105] have indicated no significant short-term toxicity of gold nanoparticles over three months. However, there are also some other studies [106] that have reported that the presence of gold nanoparticles causes cytotoxicity or inflammation in mouse livers [104]. Particularly, gold nanorods may cause cytotoxicity if they are not completely purified of surfactant CTAB. Additionally, the ideal agents in diagnosis and therapy should be completely cleared from the human body within a reasonable period. Therefore, it is essential to understand the organ uptake, biodistribution, longer-term fate, and toxicity of AuNRs, and to provide a strong framework for their clinic translation. El-Sayed et al. [88] studied the 15-month toxicity and fate of small gold nanorods in a mouse model. The histopathology of tissues from the liver, spleen, lung, and kidney of mice was evaluated by a pathologist at one month and 15 months after single intravenous injection of AuNR@PEG. There were no histopathological abnormalities in any of the mouse organs. AuNRs@PEG remained inside the cells without any structure over a long period, from visual observation of the organ tissue microstructure. During the whole treatment, gold nanorods accumulated in mouse organs without any evidence of toxicities. Similarly, Yu et al. [34] studied the size of gold nanorod impact on cytotoxicity from in vivo biodistribution. Compared to the large-sized gold nanorods (bAuNRs), the small-sized gold nanorods (sAuNRs) were cleared much more rapidly than bAuNRs (Figure 14). Therefore, small-sized gold nanorods are more suitable for in vivo imaging and tumor therapy. 

## 7. Future Challenges and Prospects

The unique surface plasma optical properties and their ultra-small size make ultra-small gold nanorods able to be widely used in the bio-imaging and cancer treatment. At the same time, ultra-small gold nanorod synthesis, surface modification, and functional applications have also made great progress. However, two aspects still need to be further improved: first, the yield of ultra-small gold nanorods needs to be improved, which may require further understanding of the process of growth of gold nanorods in solution; and, second, the extinction coefficient, which is related with the photothermal conversion efficiency, of small gold nanorods prepared by the seedless method is smaller than those prepared using the seeded technique. Thus, it is necessary to develop new methods to modify the small gold nanorods, thus, obtaining higher extinction coefficients and subsequently higher photothermal conversion efficiencies, which is of benefit to cancer treatment. With the advancement of modern science and technology, greater drawbacks for small gold nanorods will be overcome. We believe that the clinical application of small gold nanorods will be achieved in the future.
